# Assessing the global data availability and characteristics of eight risk factors for road traffic injury: an evaluation study across 194 countries/territories, 2000–2019

**DOI:** 10.7189/jogh.15.04057

**Published:** 2025-02-14

**Authors:** Wanhui Wang, Junjie Hua, David C Schwebel, Jie Li, Li Li, Zhenzhen Rao, Peixia Cheng, Peishan Ning, Guoqing Hu

**Affiliations:** 1 Department of Epidemiology and Health Statistics, Hunan Provincial Key Laboratory of Clinical Epidemiology, Xiangya School of Public Health, Central South University, Changsha City, Hunan Province, China; 2 Department of Epidemiology, School of Public Health, Sun Yat-sen University, Guangzhou City, Guangdong Province, China; 3 Department of Psychology, University of Alabama at Birmingham, Birmingham, Alabama, USA; 4 Department of Child, Adolescent and Women's Health, School of Public Health, Capital Medical University, Beijing City, China; 5 Hunan Provincial Key Laboratory of Philosophy and Social Sciences of Urban Smart Governance, Changsha City, Hunan Province, China; 6 National Clinical Research Center for Geriatric Disorders, Xiangya Hospital, Central South University, Changsha City, Hunan Province, China

## Abstract

**Background:**

Freely accessible data concerning modifiable risk factors for road traffic injury are critical for research and for evidence-based policymaking. This study investigated free-access availability and the major characteristics of nationally representative data on eight major risk factors for road traffic injury across 194 World Health Organization member countries/territories from 2000 to 2019.

**Methods:**

We systematically searched and reviewed data sources from governmental departments, multi-country road safety research projects, and international organisations. Two researchers independently searched, screened, and extracted data. We assessed free-access availability of data for eight risk factors based on the presence of data from 2000 to 2019. Major data characteristics were evaluated for all included data sources, consisting of operational definitions, method of data collection, and sampling method.

**Results:**

We identified 79 sources providing free-access available data on at least one of the eight risk factors. During 2000–2019, the number of countries/territories with freely-access data generally rose over time. However, only 134 of 194 countries/territories (69%) had at least one year of free-access data involving one or more risk factors, and 70% of those 134 countries/territories were high-income or upper middle-income countries. Large data heterogeneity existed across the data sources in terms of operational definitions used, method of data collection, years of data coverage, and sampling method. Operational definitions varied widely across the eight risk factors, ranging from 3 definitions used for fatigue driving to 17 definitions for seatbelts; and the proportion of data sources that adopted the recommended Global Road Safety Partnership (GRSP) definitions ranged from 25.5% for distracted driving to 77.8% for child restraint systems. Roadside observations were predominantly used to collect exposure data for six risk factors. Many free-access data sets were completely or partially based on non-probability sampling, and the sampling information was unknown for some additional data sources.

**Conclusions:**

Availability of free-access data on road traffic injury risks generally improved globally, but was still absent for 60 countries/territories. The substantial heterogeneity of free-access data across the existing data sources warrants further research efforts and international coordination.

According to the World Health Organization (WHO), road traffic injuries caused about 1.19 million fatalities globally in 2021 [1]. Identifying major modifiable risk factors and quantifying their health impact provides a basis for intervention development and evidence-based policymaking to address the road traffic injury challenge [2]. In 2017, the WHO released twelve voluntary global road safety performance targets for risk factors related to roads, mobility, vehicles, road users, and post-crash response [3]. These targets guide the development and practice implications of exposure measurement of major risk factors in all United Nations (UN) member states [2–4].

Valid and accurate freely-accessible data on exposure to major risk factors for road traffic injury are scarce. The most comprehensive reports released by the WHO, the Global Status Report on Road Safety (GSRRS) 2018 and 2023 address country-specific exposure data for just four risk factors (drink driving among casualties, not wearing motorcycle helmets, not using seatbelts, and not using child restraints) for 175 countries [5], and two risk factors (not wearing motorcycle helmets and not using seatbelts) for 196 countries/territories [1], respectively. The Global Burden of Disease (GBD) 2021 study group provides estimates of fatal and non-fatal road traffic injury burden attributable to six risk factors (smoking, alcohol use, low bone mineral density, occupational injuries, high temperature, and low temperature) [6]. Several developed countries regularly collect and release exposure data on other risk factors, including speeding, drink driving, drug driving, distracted driving, and fatigue driving [7–12], but these estimates are often incomparable across countries due to heterogeneous research designs and conceptualisation of the constructs being measured [13–15].

In 2020, European countries launched the ‘Baseline’ project to harmonise measurement of eight road safety indicators (speed, safety belt, protective equipment, alcohol, distraction, vehicle safety, infrastructure and post-crash care). Unfortunately, the project has not yet been fully implemented to develop key safety performance indicators, formulate data collection methods, collect data, or establish development targets for these key safety performance indicators [16,17]. In addition, several regional road safety organisations and multi-country research projects collect comparatively detailed data for common risk factors of road traffic injury, but their data are limited to the covered countries [13,18–20].

Free-access exposure data that are comparable across countries are critical for research and for evidence-based policymaking and practice at global, national, and local levels. However, no published study systematically examines free-access availability and characteristics of national data for major risk factors of road traffic injury worldwide. In response, this research examined two primary research questions:

a) what is the free-access availability of exposure data concerning eight major risk factors of road traffic injury in 194 countries/territories from 2000 to 2019?

b) did the free-access data availability about those eight major risk factors for road traffic injury change over time, across countries/territories and data sources?

## METHODS

### Selection of risk factors

Based on the WHO Global Status Report on Road Safety [1,5], we included eight risk factors for road traffic injury: speeding, drink driving, distracted driving, drug driving, fatigue driving, not wearing motorcycle helmets, not wearing seatbelts, and not using a child restraint system.

### Data sources

Free-access risk factor data were searched and derived from three types of data sources:

a) official websites of national governmental departments (typically, health, transport, and/or police departments) of 194 WHO member countries/territories;

b) websites for multi-country road safety research projects, including Social Attitudes to Road Traffic Risk in Europe (SARTRE) [19], E-Survey of Road users’ Attitudes (ESRA) [13], Driving under the Influence of Drugs, and Alcohol and Medicines (DRUID) [20], and SafetyNet, Baseline project [16]; and

c) official websites of international organisations related to road traffic safety, including Asia Injury Prevention (AIP) Foundation, International Transport Forum (ITF) [21], European Transport Safety Council (ETSC) [22], Road Safety Observatory, Institute for Health Metrics and Evaluation (IHME), Global Road Safety Partnership (GRSP) [23], United Nations, WHO, and World Bank.

Search terms used at all websites including the following words: eight core terms (speeding, drink driving, distracted driving, drug driving, fatigue driving, motorcycle helmets, seatbelts, and child restraint systems), as well as their synonyms and near-synonyms (eg, drunk driving, driving under the influence of alcohol/drug, impaired driving, mobile phone use while driving, safety belt, booster seats, safety seats).

Eligible data sources were identified according to two criteria:

a) providing national exposure data for any of the eight included risk factors;

b) derived from study samples representing the general population rather than specific populations or subpopulations.

Due to restrictions in finances, time, as well as language barriers, we only included data sources that can be accessed freely, excluding those requiring additional application and permissions, or purchase. We limited our search to data between 1 January 2000 and 31 December 2019, with no language restrictions. Data searches and screening were performed by two independent researchers and any disagreements were resolved through group discussions.

### Data collection

We extracted information from all eligible data sources, including the name of the data source, the name of the risk factor, operational definitions of the data of interest, method of data collection, sampling method, proportion of participants exposed to the risk factors, number and name of countries/territories involved, and time period with freely accessible data between 2000 and 2019. Data sources were considered nationally representative if the sample was selected at random and the collected data were used to estimate the national exposure level.

All eligible data sources were manually extracted by one trained researcher based on a standardised manual (including operational definitions and a structured table for information extraction). To ensure the quality of data extraction, we randomly selected 15% of the included data sources for re-extraction by a second researcher and obtained a consistency coefficient of 100% between the two rounds of data extractions.

### Statistical analysis

We assessed free-access availability of data using the presence of exposure data for a risk factor in the given years. Stacked bar charts were graphed to present data availability by country income, with the 194 countries/territories categorised into four income categories according to the country classification of World Bank and Asian Development Bank: 62 high-income countries (HICs), 54 upper middle-income countries (UMICs), 49 lower middle-income countries (LMICs), and 29 low-income countries (LICs) [24,25]. Following definitions recommended by the GRSP to measure road traffic injury risk factors under the voluntary global road safety targets [2,3], we calculated the proportion of data sources that adopted the recommended definitions for seven risk factors, with fatigue driving excluded because its target population was professional rather than general drivers (Table S1 in the **Online Supplementary Document**).

A heat map was plotted to display the number of countries/territories adopting different case definitions to collect exposure data for the eight risk factors between 2000 and 2019. All eligible data sources and their covered risk factors were tabulated. Major characteristics of the included data sources were displayed in detail, including method of data collection (self-report, roadside observation, both, unknown), number of years with available data (1–5 years, 6–10 years, 11–15 years, 16–20 years), and sampling method (probability, non-probability, both, partially unknown, or completely unknown).

## RESULTS

### Characteristics of included data sources

**Figure 1:**
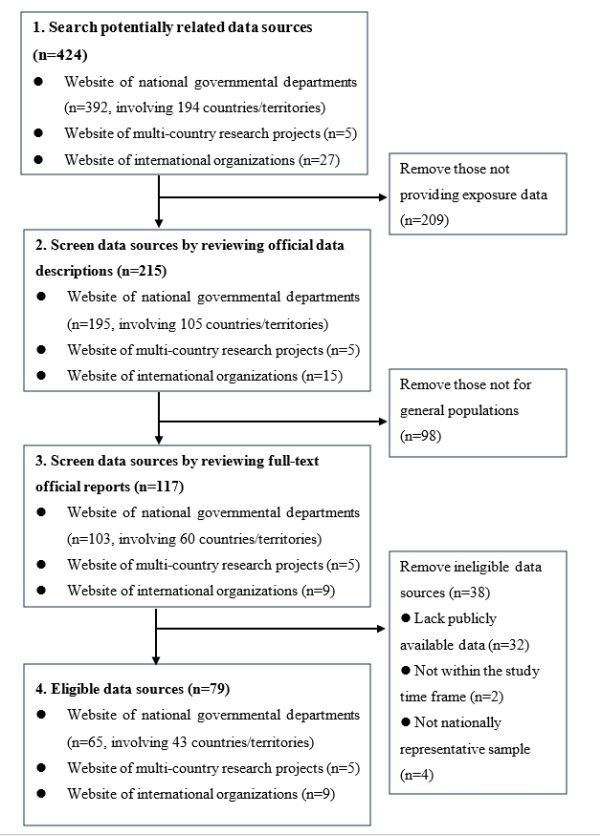
Selection of eligible data sources providing information on eight road traffic injury risk factors.

In total, 79 free-access data sources for 134 countries/territories concerning the eight risk factors of road traffic injury were included in data analysis, including 65 official websites from national governmental departments, five multi-country road safety research projects, and nine international organisations (**Figure 1**; Table S2 in the **Online Supplementary Document**).

#### Coverage of risk factors

Among these sources, 58, 45, and 45 sources respectively provide data concerning seatbelts, drink driving, and child restraint systems, and 14 concerning fatigue driving (**Table 1**). Only seven sources provide freely accessible exposure data for all eight risk factors in certain countries/territories for specific years: BFU (Beratungsstelle für Unfallverhütung, translated as Swiss Council for Accident Prevention), ESRA, International Traffic Safety Data and Analysis Group (IRTAD), Road Safety Authority of Ireland, Road Safety Authority of Serbia, SARTRE, and Vias Institute.

**Table 1:** Data availability and operational definitions used to collect data of eight road traffic injury risk factors, 2000–2019

Data source	Number of operational definitions used							
	**Speeding**	**Drink driving**	**Distracted driving**	**Drug driving**	**Fatigue driving**	**Motorcycle helmets**	**Seatbelts**	**Child restraint systems**
AAA-FTS	● (1)	● (3)	● (4)	● (2)	● (3)	ND	● (1)	ND
ACM	● (1)	ND	ND	ND	ND	ND	● (4)	● (2)
ACT	● (1)	ND	ND	ND	ND	ND	● (4)	● (1)
AIP	ND	ND	ND	ND	ND	● (3)	ND	ND
AMCPC	● (2)	ND	ND	ND	ND	ND	● (5)	● (1)
ANSR	ND	● (1)	ND	ND	ND	ND	ND	ND
ANSV	● (2)	● (2)	● (2)	ND	ND	● (3)	● (6)	● (2)
APSI	ND	ND	ND	ND	ND	ND	ND	● (1)
AVP	ND	ND	ND	ND	ND	ND	● (3)	● (1)
BASt	ND	ND	● (4)	ND	ND	● (2)	● (4)	● (1)
BFU	● (2)	● (1)	● (4)	● (2)	● (1)	● (1)	● (3)	● (1)
BITRE	● (1)	● (1)	● (1)	● (1)	ND	ND	● (1)	ND
BP	● (4)	● (6)	● (1)	ND	ND	● (7)	● (9)	● (2)
CAS	● (2)	● (1)	● (1)	ND	● (1)	ND	● (2)	ND
CCMTA	ND	● (2)	● (2)	● (3)	ND	ND	● (2)	ND
CDPC	ND	ND	ND	ND	ND	ND	● (2)	● (1)
CJIB	● (1)	ND	● (1)	ND	ND	● (1)	● (1)	ND
CONASET	ND	● (1)	● (3)	ND	ND	● (2)	● (4)	● (2)
COSEVI	ND	ND	● (1)	ND	ND	● (3)	● (4)	ND
DfT	● (2)	● (1)	● (4)	● (2)	ND	ND	● (3)	● (1)
DGT	ND	● (1)	ND	ND	ND	ND	ND	ND
DRUID	ND	● (1)	ND	● (1)	ND	ND	ND	ND
ESRA	● (1)	● (2)	● (2)	● (3)	● (2)	● (1)	● (3)	● (1)
ETSC	● (2)	● (1)	● (1)	ND	ND	● (2)	● (3)	● (1)
Federal Police of Belgium	● (1)	● (1)	● (1)	● (1)	ND	● (1)	ND	ND
FGR	ND	ND	● (1)	ND	ND	● (3)	● (4)	● (1)
Fintraffic	● (2)	ND	ND	ND	ND	ND	ND	ND
GRSP	ND	ND	ND	ND	ND	● (3)	ND	ND
GSRRS	ND	ND	ND	ND	ND	● (3)	● (4)	● (2)
I&O Research	ND	● (1)	ND	ND	ND	ND	ND	ND
IBGE	ND	● (1)	● (1)	ND	ND	● (2)	● (2)	● (1)
ICETRA	ND	● (1)	● (2)	ND	● (1)	ND	● (3)	ND
IMESEVI	ND	● (1)	ND	ND	ND	ND	● (4)	● (1)
INDEC	ND	● (1)	ND	ND	ND	● (1)	● (1)	ND
IRTAD	● (3)	● (4)	● (9)	● (3)	● (2)	● (5)	● (11)	● (4)
Italian National Police	ND	● (1)	ND	ND	ND	ND	ND	ND
ITS	● (2)	ND	● (1)	ND	ND	● (3)	● (4)	● (1)
JAF	ND	ND	ND	ND	ND	ND	● (5)	● (1)
JHLS	ND	ND	ND	ND	ND	● (3)	● (3)	ND
KfV	● (1)	ND	● (1)	ND	ND	● (1)	● (3)	● (1)
KOTSA	● (2)	● (1)	● (3)	ND	ND	● (2)	● (6)	● (1)
KSRDPR University	ND	ND	ND	ND	ND	● (2)	ND	ND
KTI	ND	ND	ND	ND	ND	ND	● (3)	● (1)
Liikenneturva	ND	ND	ND	ND	ND	ND	● (2)	ND
LiMo	● (2)	● (2)	● (2)	ND	ND	ND	● (4)	● (1)
Interior Ministry of Bulgaria	● (1)	● (1)	● (1)	ND	ND	● (1)	● (1)	ND
MIROS	● (1)	ND	ND	ND	ND	● (3)	● (4)	● (1)
MTN	ND	● (1)	ND	ND	ND	ND	ND	ND
MTNZ	● (2)	ND	● (1)	ND	● (1)	ND	● (4)	● (1)
NHTSA	● (4)	● (5)	● (7)	● (1)	ND	● (3)	● (5)	● (1)
NPAJ	● (1)	● (1)	ND	ND	ND	ND	● (1)	ND
NRSA	● (3)	● (2)	● (3)	ND	ND	ND	● (3)	● (1)
OISEVI	● (1)	● (1)	ND	ND	ND	● (3)	● (4)	● (2)
ONISR	● (2)	● (1)	● (1)	● (1)	ND	● (1)	● (2)	● (1)
PASSI	ND	● (1)	ND	ND	ND	● (1)	● (2)	● (1)
PBS	● (1)	ND	● (2)	ND	ND	ND	ND	● (1)
Police of Finland	ND	● (3)	ND	ND	ND	ND	ND	ND
Police of Slovenia	ND	● (1)	ND	ND	ND	ND	ND	ND
Police of Sweden	ND	● (1)	ND	● (1)	ND	ND	ND	ND
PSA	● (5)	ND	ND	ND	ND	ND	ND	ND
Rijkswaterstaat	ND	● (1)	● (1)	ND	ND	ND	ND	ND
RSA	● (3)	● (1)	● (5)	● (2)	● (2)	● (2)	● (9)	● (2)
RTSA	● (4)	● (2)	● (2)	● (2)	● (1)	● (3)	● (5)	● (1)
Safe Kids	ND	ND	● (2)	ND	ND	ND	ND	● (1)
SafetyNet	● (1)	ND	ND	ND	ND	● (1)	● (2)	● (1)
SARTRE	● (2)	● (3)	● (2)	● (1)	● (1)	● (1)	● (1)	● (1)
Statens vegvesen	● (1)	ND	ND	ND	ND	ND	● (4)	● (1)
STCONAPRA	● (2)	● (1)	● (2)	ND	ND	● (2)	● (1)	● (1)
SWOV	● (1)	ND	● (1)	ND	ND	ND	ND	ND
ThaiRoads Foundation	ND	ND	ND	ND	ND	● (3)	● (3)	ND
TIRF-RSM	● (1)	● (2)	● (3)	● (1)	● (2)	ND	ND	ND
Trafikverket	● (1)	● (2)	● (1)	● (2)	● (1)	ND	● (5)	ND
Transport Canada	● (1)	ND	● (1)	ND	ND	ND	● (4)	● (1)
Transportøkonomisk institutt	ND	ND	● (2)	ND	● (1)	ND	● (3)	● (1)
Ulisse	ND	ND	● (1)	ND	ND	● (1)	● (2)	ND
UNASEV	● (2)	● (2)	● (3)	● (1)	ND	● (5)	● (8)	● (2)
UNICEF	● (2)	● (2)	● (2)	ND	ND	ND	● (4)	● (1)
VIAS	● (4)	● (2)	● (3)	● (3)	● (1)	● (1)	● (7)	● (3)
YASA	ND	ND	ND	ND	ND	ND	● (2)	ND

AAA-FTS – AAA Foundation for Traffic Safety, ACM – Automobile Club of Moldova, ACT – Automobile Club of Transnistria, AIP – Asia Injury Prevention Foundation, AMCPC – Auto-Moto Organisations of the Republic of Srpska, ANSR – Autoridade Nacional Segurança Rodoviária (National Road Safety Authority), ANSV – Agencia Nacional de Seguridad Vial (National Road Safety Agency), APSI – Associação para a promoção da segurança infantil (Association for the Promotion of Child Safety), AVP – Javna agencija RS za varnost prometa (Slovenia Traffic Safety Agency), BASt – Bundesanstalt für Straßenwesen (Federal Highway Research Institute), BFU – Beratungsstelle für Unfallverhütung (Swiss Council for Accident Prevention), BITRE – Bureau of Infrastructure and Transport Research Economics, BP – Bloomberg Philanthropies, CAS – Community Attitudes Survey, CCMTA – Canadian Council of Motor Transport Administrators, CDPC – Slimību profilakses un kontroles centrs (Centre for Disease Prevention and Control of Latvia), CJIB – Centraal Justitieel Incassobureau (Central Judicial Collection Agency), CONASET – Comisión Nacional de Seguridad de Tránsito (National Commission for Traffic Safety), COSEVI – Consejo de Seguridad Vial (Road Safety Council), DfT – Department for Transport of UK, DGT – Direccion General de Trafico (General Traffic Directorate), DRUID – Driving under the Influence of Drugs, Alcohol and Medicines, ESRA – E-survey of Road users’ attitudes, ETSC – European Transport Safety Council, FGR – Fundación Gonzalo Rodríguez (Gonzalo Rodríguez Foundation), GRSP – Global Road Safety Partnership, GSRRS – Global Status Report on Road Safety, IBGE – Instituto Brasileiro de Geografia e Estatística (Brazilian Institute of Geography and Statistics), ICETRA – Icelandic Transport Authority, IMESEVI – Iniciativa Mexicana de Seguridad Vial (Mexican Road Safety Initiative), INDEC – Instituto Nacional de Estadística y Censos (National Institute of Statistics and Censuses), IRTAD – International Traffic Safety Data and Analysis Group, ITS – Instytut Transportu Samochodowego (Motor Transport Institute), JAF – Japan Automobile Federation, JHLS – Jamaica Health and Lifestyle Survey, KfV – Kuratorium für Verkehrssicherheit (Austrian Road Safety Board), KOTSA – Korea Transportation Safety Authority, KTI – Institute for Transport Sciences, LiMo – Liikluskäitumise monitooring in Estonian (Traffic Behavior Monitoring in Estonian), MIROS – Malaysian Institute of Road Safety Research, MTN – Ministry of Transport in Netherlands, MTNZ – Ministry of Transport in New Zealand, NHTSA – National Highway Traffic Safety Administration, NPAJ – National Police Agency of Japan, NRSA – National Road Safety Authority of Israel, OISEVI – Observatorio Iberoamericano de Seguridad Vial (Ibero-American Road Safety Observatory), ONISR – L'Observatoire National Interministériel de la Sécurité Routière (French Road Safety Observatory), PASSI – Progressi delle Aziende Sanitarie per la Salute in Italia (Progress of Health Companies for Health in Italy), PBS – Polish research agency, PSA – Planning and Statistics Authority, RSA – Road Safety Authority of Ireland, RTSA – Агенција за безбедност саобраћаја (Road Traffic Safety Agency of Serbia), SARTRE – Social Attitudes to Road Traffic Risk in Europe, STCONAPRA – Secretariado Técnico del Consejo Nacional para la Prevención de Accidentes (Technical Secretariat of the National Council for Accident Prevention), SWOV – Institute for Road Safety Research, TIRF-RSM – Traffic Injury Research Foundation Road Safety Monitor, UNASEV – Unidad Nacional de Seguridad Vial (National Road Safety Unit), UNICEF – United Nations International Children's Emergency Fund, VIAS – Vias Institute, YASA – Youth Association for Social Awareness.

*The number within the bracket indicated the number of operational definitions that each data source adopted to collect data.

†ND means sources that did not have relevant data.

#### Geographic coverage

Twelve data sources provide free-access data for two countries/territories or more – GSRRS (128 countries), ESRA (59), IRTAD (42), ETSC (32), SARTRE (25), SafetyNet (24), Observatorio Iberoamericano de Seguridad Vial (Ibero-American Road Safety Observatory, OISEVI) (14), DRUID (13), Bloomberg Philanthropies (BP) (9), Safe Kids (6), Fundación Gonzalo Rodríguez (Gonzalo Rodríguez Foundation, FGR) (5) and GRSP (2) (Table S2 in the **Online Supplementary Document**). The remaining 67 data sources offer freely accessible data for a single country or territory.

### Free-access availability of data

**Figure 2:**
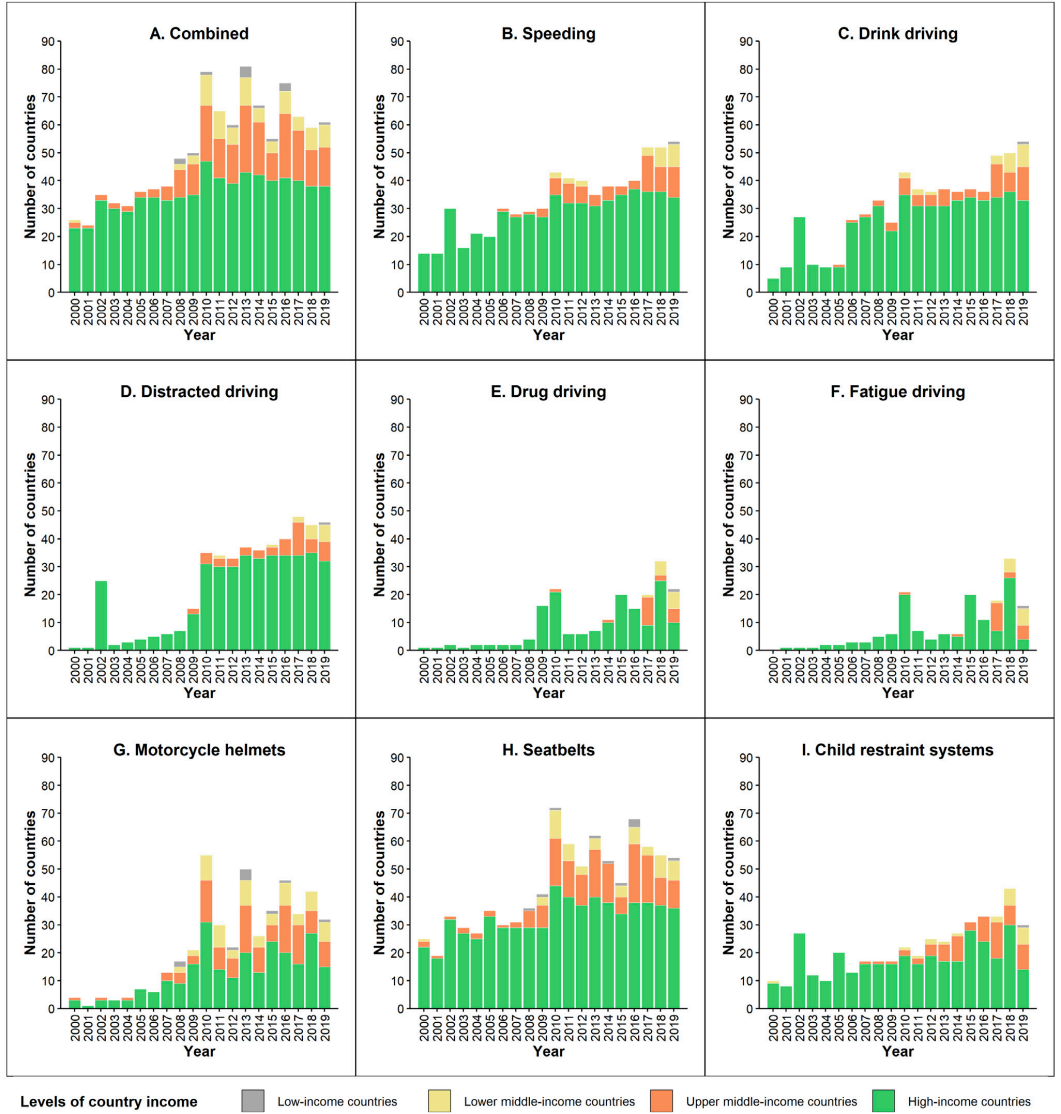
Number of countries/territories with available data on eight road traffic injury risk factors in 194 countries/territories, 2000–2019. **Panel A.** Combined. **Panel B.** Speeding. **Panel C.** Drink driving. **Panel D.** Distracted driving. **Panel E.** Drug driving. **Panel F.** Fatigue driving. **Panel G.** Motorcycle helmets. **Panel H.** Seatbelts. **Panel I.** Child restraint systems. ’Combined’ indicates the presence of any of the eight risk factors.

Out of 194 countries/territories, free-access data sources for at least one risk factors were available in 134 countries/territories (69.1%) for one year or more between 2000 and 2019 (**Figure 2**, Panel A). Global availability of free-access data generally improved over the years between 2000 and 2019, but only 60 countries/territories (30.9%) simultaneously offered freely accessible data for all eight risk factors for one year or more.

Data availability was generally much better in HICs than in the other three types of countries/territories organised by income. Most (83.9%, 52 of 62) HICs had freely accessible exposure data for one or more of the eight risk factors for at least one year; respective statistics were 77.8% (42 of 54) for UMICs, 61.2% (30 of 49) for LMICs, and 34.5% (10 of 29) for LICs. Notably, only 3.5% (1 of 29, Uganda) of LICs had freely accessible data for all eight risk factors for at least one year, compared to 50.0% (31 of 62) for HICs.

The free-access availability of exposure data varied greatly across the eight risk factors. Data were most available for seatbelts (in 122 countries/territories) and motorcycle helmets (in 116 countries/territories), and were least available for drug driving (68 countries/territories) and fatigue driving (62 countries/territories). Variations of data availability across the four types of countries/territories organised by income were roughly similar for all eight risk factors and consistently best in HICs and worst in LICs (**Figure 2**, Panels B–I).

### Major characteristics of available data

#### Operational definitions

**Figure 3:**
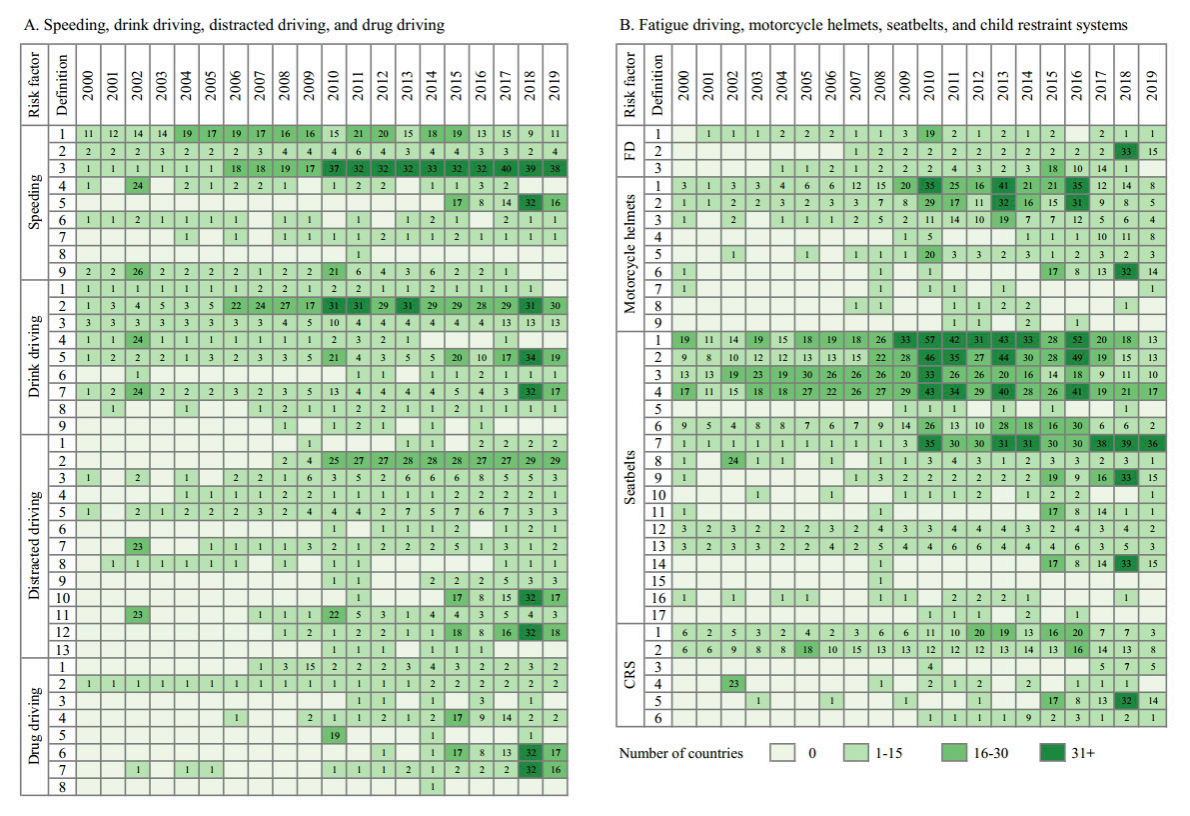
Number of countries/territories with available data adopting different definitions of eight road traffic injury risk factors, 2000-2019. **Panel A.** Speeding, drink driving, distracted driving, and drug driving. **Panel B.** Fatigue driving, motorcycle helmets, seatbelts, and child restraint systems. CRS – child restraint systems, FD – fatigue driving.

Complicating data comparisons across countries, freely accessible data for the eight risk factors were collected using inconsistent operational definitions; we identified a range from three different definitions used for fatigue driving to 17 different definitions for seatbelts (**Figure 3**; Table S3 in the **Online Supplementary Document**). The number of countries/territories adopting the same operational definition fluctuated greatly over the years between 2000 and 2019. Notably, even for the same data sources, some collected data used multiple operational definitions for the same risk factors simultaneously, especially for seatbelts, motorcycle helmets, distracted driving, and speeding.

Between 2000 and 2019, the proportion of data sources that adopted the definitions recommended by the GRSP for the seven risk factors ranged from 25.5% to 77.8%. Specific proportions include: 25.0% (11/44) for distracted driving (definition 5), 57.9% (11/19) for drug driving (definitions 1 and 4), 58.1% (25/43) for speeding (definitions 1 and 5), 65.5% (38/58) for seatbelts (definitions 1, 5, 8 and 15), 68.9% (31/45) for drink driving (definitions 2 and 5), 77.8% (35/45) for child restraint systems (definitions 2 and 6), and 78.4% (29/37) for motorcycle helmets (definitions 1 and 5) (Tables S1 and S3 in the **Online Supplementary Document**).

#### Data collection method

Exposure data for the eight risk factors were collected using varied methods. Across the eight risk factors combined, 35 sources adopted roadside observations to collect data, 15 used self-reports, and 29 employed both roadside observation and self-report (Figure S1, Table S4 in the **Online Supplementary Document**). Roadside observations were most frequently used to gather data on seatbelts (55.2%, 32/58), child restraint systems (62.2%, 28/45), and motorcycle helmets (75.7%, 28/37), while self-reports were adopted most often to collect data on fatigue driving (100%, 14/14) and drug driving (57.8%, 11/19).

#### Years of data coverage

Across the study time period of 2000 to 2019, only 34 of the 79 data sources provided free-access data over ten years or more (Figure S1 in the **Online Supplementary Document**). Specifically, the number of data sources providing ten-year data or more was 25 for seatbelts, 19 for drink driving, 15 for speeding, 12 for child restraint systems, eight for distracted driving, seven for motorcycle helmets, five for drug driving, and four for fatigue driving.

#### Sampling method

Across the eight risk factors combined, only 34.2% (27/79) of data sources used probability sampling to select study participants. 8.9% (7/79) and 13.9% (11/79) of data sources, respectively, completely or partially adopted non-probability sampling (Figure S1 in the **Online Supplementary Document**). Further, 43.0% (34/79) of data sources did not describe or incompletely described their sampling method (note that some data sources collected multiple kinds of data for the same risk factor). Among data sources that detailed their sampling methods, those using probability sampling only to select their study samples varied across the eight risk factors and was highest for seatbelts (75.0%, 27 / 36) and lowest for fatigue driving (54.5%, 7 / 11).

## DISCUSSION

### Principal findings

This study extends previous reports [5–12] to provide comprehensive evidence about the free-access availability and the characteristics of data concerning eight major road traffic injury risk factors in 194 WHO member states/territories from 2000 to 2019. We yielded four major findings. First, free-access availability of data generally improved for the eight road traffic injury risk factors from 2000 to 2019, but free-access data remained unavailable for 60 countries/territories in 2019. Second, free-access data were most available for HICs (83.9%, 52 of 62) and least available for LICs (34.5%, ten of 29), and data availability varied greatly across the eight risk factors (most frequently available for seatbelts and motorcycle helmets and least frequently for drug driving and fatigue driving). Third, heterogenous operational definitions were adopted by data sources to gather data for the same risk factors, including even in the same years and in the same countries/territories. Self-reports were used most often to collect exposure data about fatigue driving and drug driving. Last, many free-access data sets were collected completely or partially based on non-probability sampling, and some data sources failed to describe their sampling method.

### Interpretation of findings

Despite recent improvements in free-access availability of data, progress was uneven across regions and across income levels. Poor coordination among governments, inadequate financial support and restricted technological capabilities can lead to gaps in data collection and slower progress in publicising the data [26,27]. The current situation is far below ideal standards to support efforts seeking improved road traffic safety globally, particularly in most lower income countries and territories and for prevention of drug driving and fatigue driving. The poor availability of freely accessible data impinges on research, policy changes, and citizen action to understand the scope of road traffic safety in a particular country/territory and to take action to improve it.

Poor data availability can be due to multiple reasons. First, an absence of data are most common in LMICs that often lack long-term road safety planning, and that have limited funds available to invest in road infrastructure and to hire qualified personnel to collect high-quality exposure data [28–31]. Besides, social-cultural factors such as fatalistic beliefs and the desire to ‘save face’ within social networks might unintentionally suppress willingness to report surveillance data [32]. These challenges may be defensible in countries with many pressing needs, including broader public health challenges, but could be addressed through global support and optimisation of national priorities. Second, strict data protection policies are present in heavily-populated countries like India, Viet Nam, and China [5,33], all of which have collected data but decline to release them publicly, including to qualified researchers. These policies contribute to the large data availability gaps between LICs/LMICs and HIC/UMICs and could easily be reversed with changes in government policy [34,35]. Third, some risk factors are easier to measure than others. Speeding and distracted driving can be automatically detected by monitoring cameras. Drinking driving requires personnel to measure behaviour through objective strategies such as an alcohol breathalyser. Fatigue driving and drug driving are more difficult to measure efficiently and objectively, potentially impacting collection of free-access data, especially in lower-income countries [36,37].

Heterogeneity in the use of operational definitions and comparatively poor scientific rigor in data collection for some variables and countries/territories represent major challenges for policymakers, interventionists, and researchers. Our findings concord with previous reports [14] and likely reflect the combined effects of limited use of authoritative and unified operational definitions recommended by the GRSP [2,3], inadequate and unstable financial support for data collection, and absence of convenient, standard, and reliable data collection and disclosure methods. Appropriate bodies such as the WHO and GRSP should lead efforts to establish expert consensus for global standardisation, as currently no global standards or expert consensus exist to guide operational definitions and optimal data collection strategies concerning road traffic injury risk factors and other common injury causes. Financial resources are always finite but must be prioritised to support researchers and officials to implement reliable and rigorous assessment methods.

### Policy implications

Our findings underscore the importance and urgency of collecting high-quality exposure data concerning major road traffic injury risk factors to support implementation of the Decade of Action 2021–2030 [38].

We propose several solutions. First, since operational definitions and research methodologies have not been standardised globally to monitor the WHO’s voluntary global road safety performance targets [2], it would be helpful for organisations like WHO and GRSP to develop and then enforce standardised methods to guide data collection in all member states/territories. To further bolster these efforts, we recommend establishment of a global task force that comprises experts from relevant professional groups. This task force should work within the framework of existing international road safety guidelines to design, implement, and evaluate standardised methodologies. Such efforts will enhance the comparability, reliability, and utility of global road safety data, building a base to monitor the progress of road safety goals and assess the effectiveness of interventions. These standardised methods could be developed based on successful models and practices, such as the SARTRE and ESRA surveys, which adopted harmonised indicators and standardised measurement of road traffic injury risk factors to facilitate cross-country comparisons [13,19]. In addition, the DRUID project forms a helpful model, as it compiled a comprehensive manual and used it to investigate the impact of alcohol, illicit drugs, and medicines on driving in 13 European countries [20].

Second, governments worldwide should recognise the value of collecting high-quality risk factor data and identify stable long-term financial support for data collection efforts. Accurate data facilitates intervention programming and ultimately saves lives. For lower income countries that have legitimate competing urgent priorities for limited funds, foreign government and NGO support should be provided.

Third, innovative, low-cost, and convenient-to-implement assessment strategies should be developed to support data collection of difficult-to-measure risk factors like fatigue driving, distracted driving, and drug driving. Artificial intelligence-based techniques based on videos, voices, and images, such as machine learning and image processing, have potential to address the challenges of under-reporting and poor data quality by automatically capturing and analysing data, thereby minimising human involvement and associated bias [39].

Last, key data users such as policymakers, researchers, and public health practitioners should recognise both the value and potential limitations of existing freely accessible data concerning traffic safety risk factors. Efforts to compare findings across countries or years should be conducted cautiously given the varying operational definitions used to measure risks, and data collected through non-representative sampling strategies should be interpreted in the context of potential biases they may contain. For instance, leveraging the level and trend data of road safety risk factors regularly published by public databases such as the National Highway Traffic Safety Administration (NHTSA), many states in USA have enacted and continuously updated their laws to align with the latest motor vehicle safety recommendations [40]. Simultaneously, researchers should work to develop appropriate data correction methods to address methodological limitations in existing data. For instance, the WHO adopted an adjustment factor proposed by the European Conference of Ministers of Transport (ECMT) to convert road traffic death rates that were calculated based on diverse operational definitions to a standardised 30-day rate [5].

### Study limitations

This study was mainly limited by our data search strategies. We developed a series of strategies to maximally capture all potentially relevant data sources in all languages, but we might have unintentionally omitted freely accessible data presented in languages other than English or Chinese. If we did overlook a data set, however, our results would not change drastically, as our search included official websites of relevant international and regional organisations, including WHO, and those organisations previously collected data from individual governments.

In addition, because limited information appears on many official websites, we faced missing datapoints for several variables. For example, the research methods used to collect data were often not mentioned, creating a situation where the sampling methods for 43.0% of data sources (34 / 79) were completely or partially unknown. This brings unwanted uncertainty for data processing and analysis. In the future, either the WHO or another appropriate research team could conduct intensive and extensive evaluations through collaboration with relevant government bodies, multi-country research groups, and other international or regional organisations, to gather the missing datapoints and establish a standard protocol to address missing values consistently.

## CONCLUSIONS

Free-access availability of data concerning eight major road traffic injury risk factors generally improved over time in 194 countries/territories between 2000 and 2019, but relevant data were not freely accessible in 60 countries/territories, most of them from LMICs. Considerable methodological heterogeneity exists for data on the eight risk factors, including varying operational definitions used to measure constructs and varying methods of data collection, years of data coverage, and sampling strategies. Specific policy and research suggestions are recommended to address the current data challenges.

## Additional material


Online Supplementary Document
