# Supplementary material for: Assessing the global data availability and characteristics of eight risk factors for road traffic injury: an evaluation study across 194 countries/territories, 2000–2019
Source: J Glob Health. 2025 Feb 14;15:04057. doi: 10.7189/jogh.15.04057 (PMC11826958; doi:10.7189/jogh.15.04057)
Supplement: Online Supplementary Document [file jogh-15-04057-s001.pdf]

Online Supplementary Document

Table S1. Recommended definitions to measure seven road traffic injury risk factors by Global Road Safety Partnership

Table S2. List of publicly available data sources for eight road traffic injury risk factors included in the study

Table S3. Operational definitions of eight key road traffic injury risk factors adopted by the publicly accessible data sources

Figure S1. Characteristics of 79 available data sources concerning eight road traffic injury risk factors, 2000-2019

Table S4. Characteristics of available data sources on eight road traffic injury risk factors, 2000-2019

Table S1. Recommended definitions to measure seven road traffic injury risk factors by Global Road Safety Partnership

Risk factor	Method of data collection	Description of definition
Speeding	Roadside observations	Percentage of vehicle drivers complying with speed limits
	Self-reported	Percentage of vehicle drivers declaring to have been speeding in the last 30 days
Drink driving	Roadside observations	Percentage of vehicle drivers complying with alcohol driving under the influence limits
	Self-reported	Percentage of vehicle drivers declaring to have drunk alcohol over the legal limit before driving (in the last 30 days)
Distracted driving	Roadside observations	Percentage of vehicle drivers that are using their mobile phone (handheld) while driving
	Self-reported	Percentage of vehicle drivers declaring to have used their mobile phone for phoning while driving in the last 30 days
	Self-reported	Percentage of vehicle drivers declaring to have used their mobile phone for texting while driving in last 30 days
Drug driving	Roadside observations	Percentage of vehicle drivers complying with drug driving under the influence limits
	Self-reported	Percentage of vehicle drivers declaring to have used psychoactive substances before driving (in the last 30 days)
Motorcycle helmets	Roadside observations	Percentage of motorcyclists appropriately wearing an appropriate helmet
	Self-reported	Percentage of motorcyclists declaring to always wear a helmet
Seatbelts	Roadside observations	Percentage of motor vehicle drivers correctly wearing a safety belt
	Roadside observations	Percentage of motor vehicle passengers correctly wearing a safety belt
	Self-reported	Percentage of car drivers and passengers declaring to always fasten their safety belt while driving
Child restraint systems	Roadside observations	Percentage of children correctly fastened in child restraint systems (or safety belts, from the appropriate age/size)
	Self-reported	Percentage of car drivers declaring to always fasten children in an appropriate child restraint system in their car

Table S2. List of publicly available data sources for eight road traffic injury risk factors included in the study

Publicly available data source	Country/territory involved
**1. National government department**	
Agencia Nacional de Seguridad Vial (National Road Safety Agency) (ANSV)<br>Instituto Nacional de Estadística y Censos (National Institute of Statistics and Censuses) (INDEC)	Argentina
Bureau of Infrastructure and Transport Research Economics (BITRE)<br>Community Attitudes Survey (CAS)	Australia
Kuratorium für Verkehrssicherheit (Austrian Road Safety Board) (KfV)	Austria
Police Fédérale (Federal Police of Belgium)<br>Vias Institute (VIAS)	Belgium
Auto-Moto Organisations of the Republic of Srpska (AMCPC)	Bosnia and Herzegovina
Instituto Brasileiro de Geografia e Estatística (Brazilian Institute of Geography and Statistics) (IBGE)	Brazil
МИНИСТЕРСТВО НА ВЪТРЕШНИТЕ РАБОТИ (Ministry of Interior of Bulgaria)	Bulgaria
The Canadian Council of Motor Transport Administrators (CCMTA)<br>Traffic Injury Research Foundation Road Safety Monitor (TIRF-RSM)<br>Transport Canada	Canada
Comisión Nacional de Seguridad de Tránsito (National Commission for Traffic Safety) (CONASET)	Chile
Consejo de Seguridad Vial (Road Safety Council) (COSEVI)	Costa Rica
Liikluskäitumise monitooring in Estonian (Traffic Behavior Monitoring in Estonian) (LiMo)	Estonia
Fintraffic (Finnish Transport Infrastructure Agency)<br>Liikenneturva (Finnish Road Safety Council)<br>Police of Finland	Finland
L'Observatoire National Interministériel de la Sécurité Routière (French Road Safety Observatory) (ONISR)	France
Bundesanstalt für Straßenwesen (Federal Highway Research Institute) (BASt)	Germany
KTI Institute for Transport Sciences	Hungary
Icelandic Transport Authority (ICETRA)	Iceland
KSRDPR University	India
Road Safety Authority of Ireland	Ireland
National Road Safety Authority of Israel	Israel
Italian National Police<br>Progressi delle Aziende Sanitarie per la Salute in Italia (Progress of Health Companies for Health in Italy) (PASSI)<br>Ulisse	Italy
Jamaica Health and Lifestyle Survey (JHLS)	Jamaica
Japan Automobile Federation (JAF)<br>National police agency of Japan	Japan
Slimību profilakses un kontroles centrs (Centre for Disease Prevention and Control of Latvia)	Latvia
Youth Association for Social Awareness (YASA)	Lebanon
Malaysian Institute of Road Safety Research (MIROS)	Malaysia
Iniciativa Mexicana de Seguridad Vial (Mexican Road Safety Initiative) (IMESEVI)<br>STCONAPRA--Secretariado Técnico del Consejo Nacional para la Prevención de Accidentes (Technical Secretariat of the National Council for Accident Prevention)	Mexico

Publicly available data source	Country/territory involved
Centraal Justitieel Incassobureau (Central Judicial Collection Agency) (CJIB)<br>I&O Research<br>Ministry of Transport in Netherlands<br>Rijkswaterstaat Water, Verkeer en Leefomgeving (Rijkswaterstaat Water, Traffic, and Environment)<br>Institute for Road Safety Research (SWOV)	Netherlands
Ministry of Transport in New Zealand	New Zealand
Statens vegvesen (Norwegian Public Road Administration)<br>Transportøkonomisk institutt (Institute of Transport Economics)	Norway
Instytut Transportu Samochodowego (Motor Transport Institute) (ITS)<br>Polish research agency (PBS)	Poland
Associação para a promoção da segurança infantile (Association for the Promotion of Child Safety) (APSI)<br>Autoridade Nacional Segurança Rodoviária (National Road Safety Authority) (ANSR)	Portugal
Planning and Statistics Authority	Qatar
Korea Transportation Safety Authority (KOTSA)	Republic of Korea
Automobile Club of Moldova (ACM)<br>Automobile Club of Transnistria (ACT)	Republic of Moldova
Агенција за безбедност саобраћаја (Road Traffic Safety Agency of Serbia)	Serbia
Javna agencija RS za varnost prometa (Slovenia Traffic Safety Agency) (AVP)<br>Policija (Police of Slovenia)	Slovenia
Direccion General de Trafico (General Traffic Directorate) (DGT)	Spain
Polisen (Police of Sweden)<br>Trafikverket (Swedish Transport Administration)	Sweden
Beratungsstelle für Unfallverhütung (Swiss Council for Accident Prevention) (BFU)	Switzerland
ThaiRoads Foundation	Thailand
Department for Transport (DfT)	United Kingdom
AAA Foundation for traffic safety (AAA-FTS)<br>National Highway Traffic Safety Administration (NHTSA)	United States of America
Unidad Nacional de Seguridad Vial (National Road Safety Unit) (UNASEV)	Uruguay
**2. Road safety research projects**	
Bloomberg Philanthropies (BP)	9 countries/territories
Driving under the Influence of Drugs, Alcohol and Medicines (DRUID)	13 countries/territories
E-Survey of Road users' Attitudes (ESRA)	59 countries/territories
Social Attitudes to Road Traffic Risk in Europe (SARTRE)	25 countries/territories
SafetyNet	24 countries/territories
**3. International organizations**	
Asia Injury Prevention (AIP) Foundation	Cambodia
European Transport Safety Council (ETSC)	32 countries/territories
Fundación Gonzalo Rodríguez (Gonzalo Rodríguez Foundation) (FGR)	5 countries/territories
Global Road Safety Partnership (GRSP)	2 countries: India and Indonesia
International Traffic Safety Data and Analysis (IRTAD)	42 countries/territories
Observatorio Iberoamericano de Seguridad Vial (Ibero-American Road Safety Observatory) (OISEVI)	14 countries/territories
Safe Kids	6 countries/territories
United Nations International Children's Emergency Fund (UNICEF)	Montenegro
World Health Organizations - Global Status Report on Road Safety (GSRRS)	128 countries/territories

Figure S1. Characteristics of 79 available data sources concerning eight road traffic injury risk factors, 2000-2019. **Panel A.** Method of data collection. **Panel B.** Years of data coverage. **Panel C.** Sampling method.

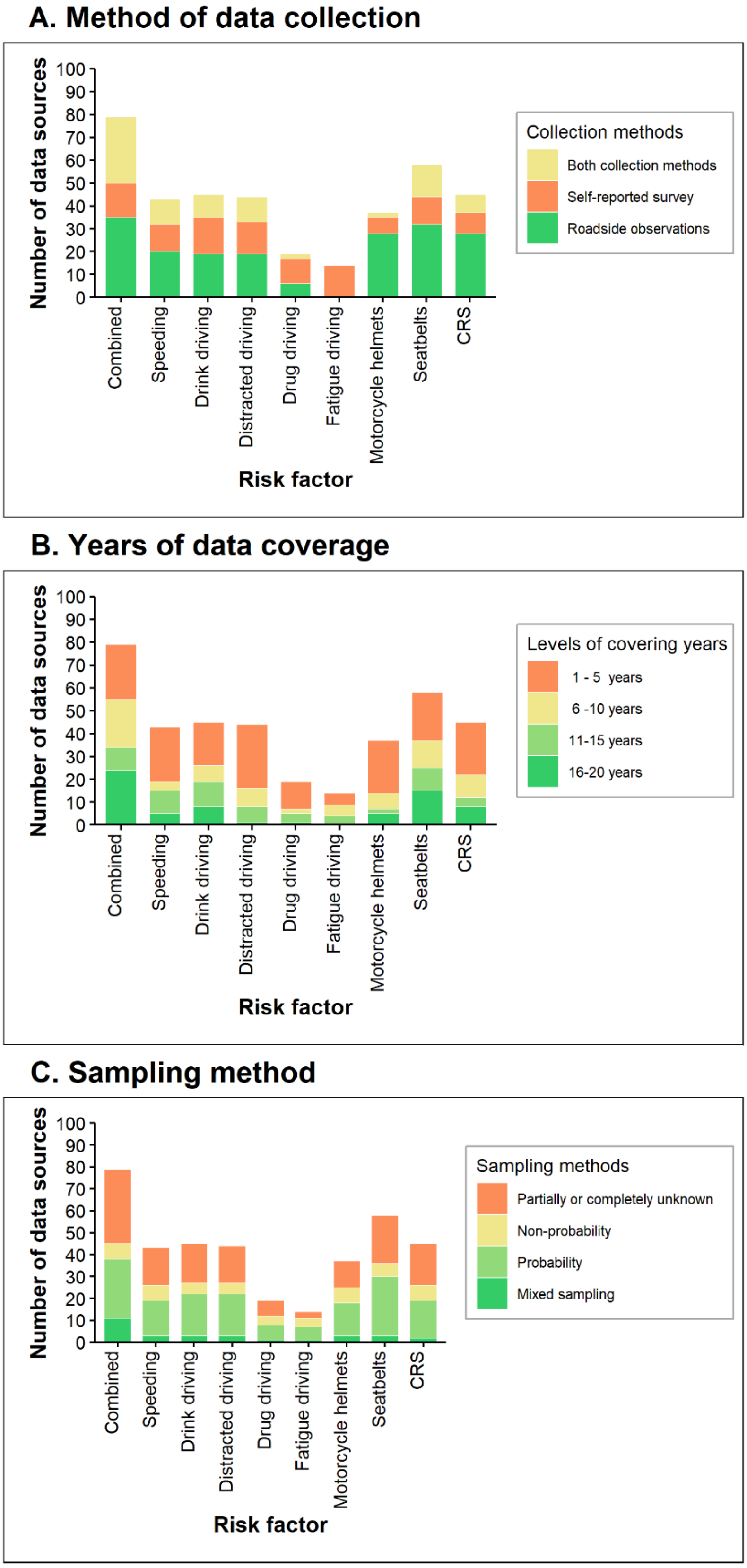

Notes: Abbreviations: CRS: Child restraint systems.

Table S3. Operational definitions of eight key road traffic injury risk factors adopted by the publicly accessible data sources

Risk factor	Type of definition	Method of data collection	Description of definition
Speeding	**Definition 1***	Roadside observations	Drive faster than speed limit
	Definition 2	Roadside observations	Drive xx km/h or more over the speed limit
	Definition 3	Roadside observations	Enforcement of speeding violations
	Definition 4	Self-reported	Drive faster than speed limit
	**Definition 5***	Self-reported	Drive faster than speed limit with time restriction
	Definition 6	Self-reported	Drive xx km/h or more over the speed limit
	Definition 7	Self-reported	Drive xx km/h or more over the speed limit in the last 30 days
	Definition 8	Self-reported	Fined or punished in any other way for breaking the speed limit
	Definition 9	Self-reported	Fined or punished in any other way for breaking the speed limit with time restriction
Drink driving	Definition 1	Roadside observations	Random breath test (Blood alcohol concentration over zero)
	**Definition 2***	Roadside observations	Random breath test (Blood alcohol concentration over the legal limit)
	Definition 3	Roadside observations	Enforcement of drink driving violations
	Definition 4	Self-reported	Drive after drinking alcohol
	**Definition 5***	Self-reported	Drive after drinking alcohol with time restriction
	Definition 6	Self-reported	Drive after drinking too much alcohol
	Definition 7	Self-reported	Drive after drinking too much alcohol with time restriction
	Definition 8	Self-reported	Drive within 2 hours after drinking alcohol with time restriction
	Definition 9	Self-reported	Fined or punished in any other way for drink driving with time restriction
Distracted driving	Definition 1	Roadside observations	Any secondary activity other than mobile phone use while driving
	Definition 2	Roadside observations	Enforcement of mobile phone use while driving violations
	Definition 3	Roadside observations	Mobile phone use while driving
	Definition 4	Roadside observations	Hands-free mobile phone use while driving
	**Definition 5***	Roadside observations	Hand-held mobile phone use while driving
	Definition 6	Self-reported	Any secondary activity other than mobile phone use while driving
	Definition 7	Self-reported	Mobile phone use while driving
	Definition 8	Self-reported	Mobile phone use while driving with time restriction
	Definition 9	Self-reported	Hands-free mobile phone use while driving
	Definition 10	Self-reported	Hands-free mobile phone use while driving with time restriction
	Definition 11	Self-reported	Hand-held mobile phone use while driving
	Definition 12	Self-reported	Hand-held mobile phone use while driving with time restriction
	Definition 13	Self-reported	Fined or punished in any other way for mobile phone use while driving
Drug driving	**Definition 1***	Roadside observations	Roadside drug test
	Definition 2	Roadside observations	Enforcement of drug driving violations

Risk factor	Type of definition	Method of data collection	Description of definition
	Definition 3	Self-reported	Drive after using illegal drugs (other than medication)
	**Definition 4***	Self-reported	Drive after using illegal drugs (other than medication) with time restriction
	Definition 5	Self-reported	Drive after using medication that may influence driving ability
	Definition 6	Self-reported	Drive after using medication that may influence driving ability with time restriction
	Definition 7	Self-reported	Drive within 1 to 2 hours after using drugs with time restriction
	Definition 8	Self-reported	Fined or punished in any other way for the use of drugs/medication while driving in the last year
Fatigue driving	Definition 1	Self-reported	Feel tired or sleepy while driving
	Definition 2	Self-reported	Feel tired or sleepy while driving in the last 30 days
	Definition 3	Self-reported	Feel tired or sleepy while driving in the last 12 months
Motorcycle helmets	**Definition 1***	Roadside observations	The driver/rider's wearing
	Definition 2	Roadside observations	The passengers' wearing
	Definition 3	Roadside observations	All occupants' wearing
	Definition 4	Roadside observations	Enforcement of not wearing motorcycle helmets violations
	**Definition 5***	Self-reported	The driver's wearing
	Definition 6	Self-reported	The driver's wearing with time restriction
	Definition 7	Self-reported	The passengers' wearing
	Definition 8	Self-reported	All occupants' wearing
	Definition 9	Self-reported	Fined or punished in any other way for not wearing a motorcycle helmet
Seatbelts	**Definition 1***	Roadside observations	The driver's wearing
	Definition 2	Roadside observations	The front seat passenger's wearing
	Definition 3	Roadside observations	Front seats' wearing (the driver and the front seat passenger)
	Definition 4	Roadside observations	Rear seats' wearing
	**Definition 5***	Roadside observations	The passengers' wearing
	Definition 6	Roadside observations	All occupants' wearing
	Definition 7	Roadside observations	Enforcement of not wearing seatbelts violations
	**Definition 8***	Self-reported	The driver's wearing
	Definition 9	Self-reported	The driver's wearing with time restriction
	Definition 10	Self-reported	The front seat passenger's wearing
	Definition 11	Self-reported	The front seat passenger's wearing in the last 12 months
	Definition 12	Self-reported	Front seats' wearing
	Definition 13	Self-reported	Rear seats' wearing
	Definition 14	Self-reported	Rear seats' wearing with time restriction
	**Definition 15***	Self-reported	The passengers' wearing
	Definition 16	Self-reported	All occupants' wearing
	Definition 17	Self-reported	Fined or punished in any other way for not wearing seatbelts while driving
Child restraint systems	Definition 1	Roadside observations	Children
	**Definition 2***	Roadside observations	Children by age group

Risk factor	Type of definition	Method of data collection	Description of definition
	Definition 3	Roadside observations	Enforcement of not wearing child restraint systems violations
	Definition 4	Self-reported	Children
	Definition 5	Self-reported	Children under xx cm with time restriction
	**Definition 6***	Self-reported	Children by age group

*Operational definitions as global road safety performance indicators recommended by the Global Road Safety Partnership.

Table S4. Characteristics of available data sources on eight road traffic injury risk factors, 2000-2019

Data source	Method of data collection	Covering years of data	Countries/territories being covered	Sampling method	Notes
AAA-FTS	Self-reported	2008-2018	United States of America	Probability	
ACM	Both	2012	Republic of Moldova	Non-probability	
ACT	Both	2012	Republic of Moldova	Non-probability	
AIP	Both	2011, 2014, and 2016	Cambodia	Probability	
AMCPC	Roadside observations	2012-2019	Bosnia and Herzegovina	Probability	
ANSR	Roadside observations	2010-2019	Portugal	Probability	
ANSV	Both	2011-2014, and 2016-2018	Argentina	Both probability and non-probability	The survey of drink driving between 2016 and 2018 was non-probability sampling and not nationally representative.
APSI	Roadside observations	2001-2018	Portugal	N/A	
AVP	Roadside observations	2005,2007-2011,2016	Slovenia	Part probability	
BASt	Both	2000-2019	Germany	Both probability and non-probability	Representative of urban areas
BFU	Both	2000-2019	Switzerland	Part probability	
BITRE	Roadside observations	2008-2019	Australia	N/A	
GRSP	Both	2007 and 2011	India and Indonesia	Non-probability	In selected cities
CAS	Self-reported	2000-2006, 2008-2009, 2011, 2013 and 2017	Australia	Probability	
CCMTA	Both	2007, 2010, 2012-2013 and 2016-2018	Canada	Probability	(1) Rural results for year of 2006, 2009, 2013 and 2017; urban results for 2007, 2010, 2012 and 2016, allowing nationally representative data to be obtained from data from two consecutive years. (2) the study year was the year used to report nationally representative data.
CDPC	Self-reported	Every two years from 2000 to 2018	Latvia	Probability	
CJIB	Roadside observations	2017-2019	Netherlands	N/A	
CONASET	Both	2013-2019	Chile	Part probability	In Gran Santiago

Data source	Method of data collection	Covering years of data	Countries/territories being covered	Sampling method	Notes
COSEVI	Roadside observations	2014, 2016 and 2018	Costa Rica	N/A	
DfT	Both	2000-2019	United Kingdom	Part probability	
DGT	Roadside observations	2011-2019	Spain	N/A	
FGR	Roadside observations	2013-2017	Argentina, Chile, Colombia, Paraguay, Uruguay	N/A	Aggregated second-hand data
Safe Kids	Both	2008-2009, and 2013-2014	Brazil, China, India, Qatar, South Africa and United States of America	Part non-probability	The estimation of CRS was in active school zones
BP	Both	2009-2014	Cambodia, China, Egypt, India, Kenya, Mexico, Russia Federation, Türkiye and Viet Nam	Part probability	In selected provinces or cities
Federal Police of Belgium	Roadside observations	2008-2019	Belgium	N/A	
DRUID	Roadside observations	2009	Belgium, Czechia, Denmark, Finland, Hungary, Italy, Lithuania, Netherlands, Norway, Poland, Portugal, Spain, Sweden	Probability	
Fintraffic	Roadside observations	2010-2019	Finland	Probability	
OISEVI	Roadside observations	2010, 2012, 2014, 2016-2019	Andorra, Argentina, Chile, Colombia, Costa Rica, Dominican Republic, Guatemala, Honduras, Mexico, Nicaragua, Paraguay, Portugal, Spain, Uruguay	N/A	Aggregated second-hand data
SafetyNet	Roadside observations	2002, 2005 and 2007	Austria, Belgium, Bulgaria, Czechia, Denmark, Estonia, Finland, France, Germany, Hungary, Ireland, Italy, Latvia, Luxembourg, Malta, Netherlands, Norway, Poland, Portugal, Slovenia, Spain, Sweden, Switzerland, United Kingdom of Great Britain and Northern Ireland	N/A	Aggregated second-hand data
I&O Research	Roadside observations	2010-2011, 2013, 2015, 2017 and 2019	Netherlands	Probability	
IBGE	Self-reported	2013, 2015 and 2019	Brazil	Probability	
ICETRA	Self-reported	2007-2019	Iceland	Probability	
IMESEVI	Roadside observations	2008-2010	Mexico	Probability	In selected cities

Data source	Method of data collection	Covering years of data	Countries/territories being covered	Sampling method	Notes
INDEC	Self-reported	2005, 2009, 2013 and 2018	Argentina	Probability	Representative of urban areas
SARTRE	Self-reported	2002 and 2010	Austria, Belgium, Croatia, Cyprus, Czechia, Denmark, Estonia, Finland, France, Germany, Greece, Hungary, Ireland, Israel, Italy, Netherlands, Poland, Portugal, Serbia, Slovakia, Slovenia, Spain, Sweden, Switzerland, United Kingdom of Great Britain and Northern Ireland	Both probability and non-probability	Some countries conducted quota sampling while others conducted random sampling.
Italian National Police	Roadside observations	2006-2010	Italy	N/A	
ITS	Roadside observations	2004, 2006, 2008, 2013-2015	Poland	Both probability and non-probability	
JAF	Both	2002-2019	Japan	Both probability and non-probability	
JHLS	Self-reported	2000, 2008 and 2017	Jamaica	Probability	
KFV	Roadside observations	2000-2019	Austria	Probability	
KOTSA	Both	2000-2007, 2010-2019	Republic of Korea	Both probability and non-probability	
KSRDPR University	Roadside observations	2017	India	Probability	In Bangalore
KTI	Roadside observations	2000-2005, 2007-2009	Hungary	N/A	
Liikenneturv a	Roadside observations	2000-2019	Finland	Probability	Result consists of two parts (urban areas and outside urban areas), no summary value
LiMo	Both	2000-2019	Estonia	Part probability	In selected sites or cities
Ministry of Interior of Bulgaria	Roadside observations	2009	Bulgaria	N/A	
MIROS	Roadside observations	2003, 2008-2019	Malaysia	Part non-probability	In selected cities
MTN	Self-reported	2000-2016	Netherlands	Probability	
MTNZ	Both	2000-2016	New Zealand	Part probability	
NHTSA	Both	2000-2019	United States of America	Probability	
NPAJ	Roadside observations	2000-2019	Japan	N/A	
NRSA	Both	2005-2019	Israel	Part probability	

Data source	Method of data collection	Covering years of data	Countries/territories being covered	Sampling method	Notes
ETSC	Roadside observations	2000-2019	Austria, Belgium, Bulgaria, Croatia, Cyprus, Czechia, Denmark, Estonia, Finland, France, Germany, Greece, Hungary, Ireland, Israel, Italy, Latvia, Lithuania, Luxembourg, Malta, Netherlands, Norway, Poland, Portugal, Romania, Serbia, Slovakia, Slovenia, Spain, Sweden, Switzerland, United Kingdom of Great Britain and Northern Ireland	N/A	Aggregated second-hand data
ONISR	Roadside observations	2000-2019	France	Part probability	
PASSI	Self-reported	2007-2019	Italy	Non-probability	
PBS	Self-reported	2014	Poland	Non-probability	In selected areas
Police of Finland	Roadside observations	2000-2019	Finland	N/A	
Police of Slovenia	Roadside observations	2001-2016	Slovenia	N/A	
Police of Sweden	Roadside observations	2000-2019	Sweden	N/A	
PSA	Roadside observations	2014-2019	Qatar	N/A	
Rijkswaterstaat	Roadside observations	2002, 2005-2008, 2016 and 2018	Netherlands	Probability	
RSA	Both	2002-2009, 2011-2019	Ireland	Both probability and non-probability	
RTSA	Both	2013-2019	Serbia	Both probability and non-probability	
IRTAD	Both	2000-2019	Argentina, Australia, Austria, Belgium, Cambodia, Canada, Chile, Colombia, Czechia, Denmark, Finland, France, Germany, Greece, Hungary, Iceland, Ireland, Israel, Italy, Jamaica, Japan, Lithuania, Luxembourg, Malaysia, Mexico, Morocco, Netherlands, New Zealand, Nigeria, Norway, Poland, Portugal, Republic of Korea, Serbia, Slovenia, South Africa, Spain, Sweden, Switzerland, United Kingdom of Great Britain and Northern Ireland, United States of America, Uruguay	Both probability and non-probability	Aggregated second-hand data

Data source	Method of data collection	Covering years of data	Countries/territories being covered	Sampling method	Notes
ESRA	Self-reported	2015-2019	Argentina, Australia, Austria, Belgium, Benin, Bolivia (Plurinational State of), Brazil, Bulgaria, Cameroon, Canada, Chile, Colombia, Costa Rica, Czechia, Denmark, Ecuador, Egypt, Finland, France, Germany, Ghana, Greece, Guatemala, Hungary, Iceland, India, Ireland, Israel, Italy, Japan, Kenya, Lebanon, Luxembourg, Malaysia, Mexico, Morocco, Netherlands, Nigeria, Norway, Paraguay, Peru, Poland, Portugal, Republic of Korea, Serbia, Slovenia, South Africa, Spain, Sweden, Switzerland, Thailand, Tunisia, Uganda, United Kingdom of Great Britain and Northern Ireland, United States of America, Uruguay, Venezuela (Bolivarian Republic of), Viet Nam, Zambia	Non-probability	(1) The first edition of the ESRA survey was carried out in three waves in 2015, 2016 and 2017, and the second edition in two waves in 2018 and 2019. (2) Quota sampling. (3) National representativeness of some countries is affected by internet penetration and sample characteristics.
GSRRS	Both	2002-2004, 2006-2018	Albania, Algeria, Andorra, Angola, Argentina, Armenia, Australia, Austria, Azerbaijan, Bahrain, Belgium, Belize, Benin, Bolivia (Plurinational State of), Bosnia and Herzegovina, Botswana, Brazil, Brunei Darussalam, Bulgaria, Burkina Faso, Cambodia, Canada, Chile, China, Colombia, Congo, Costa Rica, Cote d'Ivoire, Croatia, Cuba, Cyprus, Czechia, Denmark, Dominican Republic, Ecuador, Egypt, Eritrea, Estonia, Eswatini, Ethiopia, Fiji, Finland, France, Georgia, Germany, Ghana, Greece, Guatemala, Guinea, Guyana, Hungary, Iceland, India, Indonesia, Iran (Islamic Republic of), Ireland, Israel, Italy, Jamaica, Japan, Jordan, Kenya, Lao People's Democratic Republic, Latvia, Lebanon, Lesotho, Lithuania, Luxembourg, Madagascar, Malawi, Malaysia, Mali, Malta, Marshall Islands, Mauritius, Mexico, Monaco, Mongolia, Montenegro, Morocco, Myanmar, Namibia, Netherlands, New	N/A	(1) Aggregated second-hand data; (2) Theses sources contain aggregated data sources, including self-report surveys, roadside observations, and modelling estimations

Data source	Method of data collection	Covering years of data	Countries/territories being covered	Sampling method	Notes
			Zealand, Nigeria, North Macedonia, Norway, Oman, Pakistan, Panama, Paraguay, Peru, Philippines, Poland, Portugal, Qatar, Republic of Korea, Republic of Moldova, Romania, Russian Federation, Saint Kitts and Nevis, Saint Lucia, Samoa, San Marino, Senegal, Serbia, Seychelles, Slovenia, South Africa, Spain, Sri Lanka, Suriname, Sweden, Switzerland, Syrian Arab Republic, Thailand, Tonga, Trinidad and Tobago, Türkiye, Uganda, United Arab Emirates, United Kingdom of Great Britain and Northern Ireland, United States of America, Uruguay, Vanuatu, Venezuela (Bolivarian Republic of), Viet Nam, Yemen		
Statens vegvesen	Both	2000-2017	Norway	Probability	
STCONAPR A	Both	2009-2019	Mexico	Both probability and non-probability	
SWOV	Both	2016-2019	Netherlands	Part probability	
ThaiRoads Foundation	Roadside observations	2010-2019	Thailand	Probability	
TIRF-RSM	Self-reported	2001-2019	Canada	Probability	
Trafikverket	Both	2000-2019	Sweden	Part probability	
Transport Canada	Both	2000-2007 and 2009-2010	Canada	Probability	Rural representative data estimated in one year and urban representative data in the following year, so nationally representative data were obtained from data across two consecutive years
Transportøk onomisk institutt	Self-reported	2000, 2002, 2004, 2008, 2011 and 2016	Norway	Probability	
Ulisse	Roadside observations	2002 (survey 2000-2002), 2005 (survey 2003-2005), and 2009-2011	Italy	Non-probability	

Data source	Method of data collection	Covering years of data	Countries/territories being covered	Sampling method	Notes
UNASEV	Both	2011-2014 and 2016	Uruguay	Part non-probability	
UNICEF	Self-reported	2010	Montenegro	Probability	
VIAS	Both	2003-2013, 2015 and 2018	Belgium	Both probability and non-probability	
YASA	Roadside observations	2001-2002, 2005, 2009, 2013-2014 and 2017	Lebanon	N/A	Representative of the five major Lebanese regions.

Abbreviations:
AAA-FTS – AAA Foundation for Traffic Safety, ACM – Automobile Club of Moldova, ACT – Automobile Club of Transnistria, AIP – Asia Injury Prevention Foundation, AMCPC – Auto-Moto Organisations of the Republic of Srpska, ANSR – Autoridade Nacional Segurança Rodoviária (National Road Safety Authority), ANSV – Agencia Nacional de Seguridad Vial (National Road Safety Agency), APSI – Associação para a promoção da segurança infantil (Association for the Promotion of Child Safety), AVP – Javna agencija RS za varnost prometa (Slovenia Traffic Safety Agency), BASt – Bundesanstalt für Straßenwesen (Federal Highway Research Institute), BFU – Beratungsstelle für Unfallverhütung (Swiss Council for Accident Prevention), BITRE – Bureau of Infrastructure and Transport Research Economics, BP – Bloomberg Philanthropies, CAS – Community Attitudes Survey, CCMTA – Canadian Council of Motor Transport Administrators, CDPC – Slimību profilakses un kontroles centrs (Centre for Disease Prevention and Control of Latvia), CJIB – Centraal Justitieel Incassobureau (Central Judicial Collection Agency), CONASET – Comisión Nacional de Seguridad de Tránsito (National Commission for Traffic Safety), COSEVI – Consejo de Seguridad Vial (Road Safety Council), DfT – Department for Transport of UK, DGT – Direccion General de Trafico (General Traffic Directorate), DRUID – Driving under the Influence of Drugs, Alcohol and Medicines, ESRA – E-survey of Road users' attitudes, ETSC – European Transport Safety Council, FGR – Fundación Gonzalo Rodríguez (Gonzalo Rodríguez Foundation), GRSP – Global Road Safety Partnership, GSRRS – Global Status Report on Road Safety, IBGE – Instituto Brasileiro de Geografia e Estatística (Brazilian Institute of Geography and Statistics), ICETRA – Icelandic Transport Authority, IMESEVI – Iniciativa Mexicana de Seguridad Vial (Mexican Road Safety Initiative), INDEC – Instituto Nacional de Estadística y Censos (National Institute of Statistics and Censuses), IRTAD – International Traffic Safety Data and Analysis Group, ITS – Instytut Transportu Samochodowego (Motor Transport Institute), JAF – Japan Automobile Federation, JHLS – Jamaica Health and Lifestyle Survey, KfV – Kuratorium für Verkehrssicherheit (Austrian Road Safety Board), KOTSA – Korea Transportation Safety Authority, KTI – Institute for Transport Sciences, LiMo – Liikluskäitumise monitooring in Estonian (Traffic Behavior Monitoring in Estonian), MIROS – Malaysian Institute of Road Safety Research, MTN – Ministry of Transport in Netherlands, MTNZ – Ministry of Transport in New Zealand, NHTSA – National Highway Traffic Safety Administration, NPAJ – National Police Agency of Japan, NRSA – National Road Safety Authority of Israel, OISEVI – Observatorio Iberoamericano de Seguridad Vial (Ibero-American Road Safety Observatory), ONISR – L'Observatoire National Interministériel de la Sécurité Routière (French Road Safety Observatory), PASSI – Progressi delle Aziende Sanitarie per la Salute in Italia (Progress of Health Companies for Health in Italy), PBS – Polish research agency, PSA – Planning and Statistics Authority, RSA – Road Safety Authority of Ireland, RTSA – Агенција за безбедност саобраћаја (Road Traffic Safety Agency of Serbia), SARTRE – Social Attitudes to Road Traffic Risk in Europe, STCONAPRA – Secretariado Técnico del Consejo Nacional para la Prevención de Accidentes (Technical Secretariat of the National Council for Accident Prevention), SWOV – Institute for Road Safety Research, TIRF-RSM – Traffic Injury Research Foundation Road Safety Monitor, UNASEV – Unidad Nacional de Seguridad Vial (National Road Safety Unit), UNICEF – United Nations International Children's Emergency Fund, VIAS – Vias Institute, YASA – Youth Association for Social Awareness.

Notes: * N/A means relevant information cannot be obtained through online search. Both refers to both self-reported and roadside observations.
